# Multi-Omics Integration Reveals Synergistic Metabolic Rewiring Underpinning Growth Acceleration in a Hybrid Pompano “Chenhai No. 1”

**DOI:** 10.3390/ani16121895

**Published:** 2026-06-18

**Authors:** Hongxuan Liang, Xin Gao, Zhennian Chen, Lang Qin, Can Xu, Yingying Yang, Yuxiang Wang, Fangzhou Hu, Xu Huang, Chang Wu, Shaojun Liu

**Affiliations:** 1 Engineering Research Center of Polyploid Fish Reproduction and Breeding of the State Education Ministry, College of Life Sciences, Hunan Normal University, Changsha 410081, China; l1478887183@163.com (H.L.);; 2 Yuelushan Laboratory, Changsha 410082, China; 3 School of Life and Environmental Sciences, Hunan University of Arts and Science, Changde 415000, China; 4 Laboratory of Tropical Marine Fish Germplasm Innovation and Utilization, Ministry of Agriculture and Rural Affairs, Hainan Engineering Research Center for Germplasm Innovation and Utilization, Hainan Chenhai Institute of Fishery Sciences, Sanya 572025, China; 5 Key Laboratory of Tropical Aquatic Germplasm of Hainan Province (MOE), Key Laboratory of Marine Genetics and Breeding, College of Marine Life Sciences, Sanya Oceanographic Institution, Ocean University of China, Sanya 572025, China

**Keywords:** hybrid golden pompano, transcriptomics, metabolomics, growth regulation, energy metabolism, mTOR signaling

## Abstract

The hybrid golden pompano “Chenhai No. 1” (CH), generated through distant hybridization [(♀ *Trachinotus ovatus* × ♂ *T. blochii*) × ♂ *T. ovatus*], exhibits significantly enhanced growth performance compared to its parental *T. ovatus* (TO). To elucidate the molecular mechanisms underlying this rapid growth, we performed integrated transcriptomic and untargeted metabolomic profiling of muscle tissue. Transcriptomic analysis identified 3172 differentially expressed genes (DEGs), with weighted gene co-expression network analysis (WGCNA) highlighting the ‘darkorange2’ module as strongly associated with rapid growth. Key DEGs, including *mapk8a*, *acacb*, and *pkmb*, were upregulated and implicated in energy metabolism, glycolysis, and signal transduction. Metabolomic profiling detected 576 significantly altered metabolites, predominantly enriched in glycolysis, the tricarboxylic acid (TCA) cycle, amino acid metabolism, lipid biosynthesis, and mTOR signaling. Integrated analysis revealed coordinated alterations between core module genes and differential metabolites in interrelated pathways, including correlations between *pfkpa*/*pfkma* and glyceraldehyde-3-phosphate, *acacb* and phosphatidylcholine/phosphatidylethanolamine, and *sesn2* and leucine. These findings suggest that the growth advantage of CH arises from the coordinated enhancement of energy metabolism, amino acid sensing, and lipid metabolic remodeling, establishing a synergistic transcription–metabolism regulatory network. This study provides multi-omics insights into the molecular basis of rapid growth in an economically important teleost fish.

## Simple Summary

The hybrid golden pompano “Chenhai No. 1” (CH) exhibits faster growth than its parental species, yet the underlying mechanisms remain to be clarified. This study employed integrated transcriptomic and metabolomic analyses of muscle tissue to investigate this growth advantage. The findings indicate that CH exhibits enhanced energy metabolism, particularly in glycolysis and the tricarboxylic acid cycle, which provides sufficient energy to support rapid growth. Increased levels of amino acids, notably leucine, suggest enhanced protein synthesis and nutrient sensing. Elevated lipid synthesis was also observed, potentially supporting cell growth and tissue development. These metabolic alterations are closely associated with the activation of the mTOR signaling pathway. Collectively, the coordinated regulation of energy production, amino acid metabolism, and lipid biosynthesis likely contributes to the rapid growth of CH.

## 1. Introduction

Aquaculture plays a critical role in supplying high-quality protein and supporting global food security [1]. However, many commercially important aquaculture species remain at an early stage of domestication, and their genetic improvement still relies heavily on wild populations [2]. Consequently, the development of novel germplasm resources with superior growth performance has become a central objective in modern aquaculture breeding programs.

Distant hybridization is an effective strategy for germplasm innovation, as it enables the integration of genomes from different species and generates extensive genetic variation [3,4,5]. This approach has been successfully applied to produce multiple aquaculture varieties with improved growth, survival, and environmental adaptability [6,7,8]. Recently, a novel hybrid golden pompano, “Chenhai No. 1” (2*n* = 48), was generated through distant hybridization between *Trachinotus ovatus* (♀) and *T. blochii* (♂), followed by backcrossing with *T. ovatus* [9]. In a feeding trial conducted from May to August, with 50 individuals per group randomly sampled for each weighing, Chenhai No. 1 exhibited superior growth performance compared with *T. ovatus*. Specifically, the final body weight of Chenhai No. 1 in August reached 579.87 ± 36.98 g, which was 35.52% higher than that of *T. ovatus* (427.88 ± 23.60 g) [9]. Nevertheless, the molecular mechanisms responsible for its growth superiority have not yet been elucidated. For convenience, Chenhai No. 1 and T. ovatus are hereinafter abbreviated as CH and TO, respectively.

In fish, skeletal muscle serves as the primary tissue driving growth [10], and its development and growth rate directly affects body weight gain, feed efficiency, and overall aquaculture productivity. These relationships have been extensively confirmed through multi-omics analyses at both the transcriptomic and metabolic levels [11]. Similarly to mammals, the growth of fish skeletal muscle typically involves both an increase in muscle fiber number and an enlargement of fiber size [12]. Numerous studies indicate that, during the growth and development of most teleost fish, hyperplasia and hypertrophy dominate at different developmental stages or in distinct tissues, and are jointly modulated by genetic regulation, nutritional input, and energy metabolism [13,14,15]. Therefore, deciphering the molecular regulatory mechanisms within muscle tissue is a crucial strategy for understanding rapid growth and improving desirable traits in fish.

Transcriptomics enables the comprehensive profiling of gene expression across all genes in a cell or tissue under specific conditions, whereas metabolomics captures the accumulation and dynamics of small-molecule metabolites within an organism [16]. By providing insights into biological states at both the gene expression and metabolite levels, integrated transcriptomic and metabolomic analyses have been extensively applied to uncover diverse molecular regulatory mechanisms [17]. For instance, integrated transcriptomic and metabolomic analyses of muscle tissues from fast- and slow-growing barramundi have identified key pathways and candidate molecules involved in muscle growth regulation [10]. Similarly, in studies on large yellow croaker (*Larimichthys crocea*), such integrative analyses have elucidated the systemic molecular mechanisms governing growth and development at the pathway level [18]. Despite these advances, the molecular mechanisms underlying the growth advantage of hybrid golden pompano, particularly the cultivar “Chenhai No. 1”, are still not fully understood. Notably, integrative transcriptomic and metabolomic analyses focused on muscle tissue are still lacking in terms of interpreting its rapid growth traits. Therefore, this study aimed to address this research gap and explore the molecular basis of the growth superiority of CH.

To investigate the molecular basis underlying the rapid growth of CH, integrated transcriptomic and untargeted metabolomic analyses of muscle tissue were performed to identify key regulatory pathways associated with growth superiority.

## 2. Materials and Methods

### 2.1. Ethics Statement

According to relevant regulations, ethical approval from the Science and Technology Bureau of China and the Department of Wildlife Administration is not required for studies involving fish species that are not classified as rare or endangered (i.e., not under first- or second-class state protection). All experimental procedures were reviewed and approved by the Biomedical Research Ethics Committee of Hunan Normal University (approval number: 2023 No. 610). Prior to sampling, all procedures were performed to minimize animal suffering. All personnel involved in the experiments received professional training and were certified by the Institute of Experimental Animals, Hunan Province, China.

### 2.2. Sample Collection

The experimental fish were reared at Chenhai Aquaculture Co., Ltd., Lingshui, China. A total of 2000 juveniles per group (TO and CH), with an initial body length of 7–8 cm, were stocked in seawater pond cages (6 × 6 × 6 m). At this early developmental stage, sex could not be reliably distinguished and was therefore not determined. Both groups were reared under identical conditions within the same pond system and were fed twice daily (morning and evening) to apparent satiation with a commercial diet (“Yuequanyuan” brand; Guangdong Yuequn Marine Biotechnology Co., Ltd., Jieyang, China).

As described in our previous study [9] and summarized in Table 1, a feeding trial was conducted from May to August during which 50 individuals per group were randomly sampled for each weighing. The growth performance results are presented below.

At the end of this 120-day growth trial, six individuals from each group were randomly selected from the respective pond populations. All fish were fasted for 24 h prior to sampling. Then, they were sampled for omics analyses. Fish were anesthetized with 80 mg/L MS-222 prior to tissue collection. Scales from the dorsal region were removed, and dorsal muscle tissue was collected. The samples were transferred into RNase-free EP tubes, clearly labeled, immediately frozen in liquid nitrogen, and stored at −80 °C for subsequent analyses.

### 2.3. Transcriptome Sequencing and Analysis

For transcriptomic analysis, three individuals were randomly selected from each group (TO and CH), yielding a total of six samples. Total RNA was extracted from dorsal muscle tissues using an RNA extraction kit (Sangon Biotech, Shanghai, China). The integrity and quality of RNA were evaluated by capillary electrophoresis with an Agilent Bioanalyzer (Agilent Technologies, Santa Clara, CA, USA) to ensure suitability for sequencing. Transcriptome library construction, high-throughput sequencing, and initial data processing were conducted by OE Biotech Co., Ltd. (Shanghai, China).

The raw sequencing data generated from high-throughput sequencing were in FASTQ format. Quality control of the raw reads was performed using Trimmomatic 0.39 [19] to remove adapters and low-quality bases, yielding clean reads for downstream analyses. De novo transcriptome assembly was carried out using Trinity [20] (version 2.4), and the longest transcript of each gene was selected as a Unigene for functional annotation and expression profiling. Assembly completeness was assessed using BUSCO (Benchmarking Universal Single-Copy Orthologs, v5.4.4) with the Actinopterygii lineage dataset (odb10, containing 303 conserved single-copy orthologs). BUSCO was run in transcriptome mode with default parameters. The percentages of complete, duplicated, fragmented, and missing orthologs were calculated to evaluate assembly quality. Functional annotation of Unigene sequences was performed via BLAST+ 2.12.0 [21] against the NR, COG/KOG, and Swiss–Prot databases with an E-value threshold of <1 × 10^−5^. Clean reads were then mapped back to the Unigenes using Bowtie 2.3.3.1 [22], and read counts and FPKM (fragments per kilobase of transcript per million mapped reads) values were calculated with eXpress [23]. Differential expression analysis was performed using the DESeq2 (version 1.20.0) [24] with normalization performed using the estimateSizeFactors function. Raw *p*-values were adjusted for multiple testing using the Benjamini–Hochberg method, and the resulting q-values (false discovery rate, FDR) were used to assess statistical significance. Genes with |log2 fold change| ≥ 1 and FDR < 0.05 were considered significantly differentially expressed.

Weighted gene co-expression network analysis (WGCNA) was performed based on the gene expression matrix. Genes with low expression variance (SD ≤ 0.5) were excluded, resulting in a set of 9064 genes for analysis. An unsigned scale-free network was constructed using a soft-threshold power of 20. Co-expression modules were identified through topological overlap matrix (TOM)-based hierarchical clustering combined with the dynamic tree cut method, with highly similar modules merged and the gray module excluded. Module eigengenes (MEs) were then correlated with sample traits using Pearson correlation, and modules with |r| ≥ 0.3 and *p* < 0.05 were considered significant. Core genes within these significant modules were subsequently identified, and KEGG pathway enrichment analysis was conducted to investigate the potential biological functions and regulatory pathways associated with each module.

### 2.4. Metabolomics Analysis by LC–MS and GC–MS

A total of 12 samples were used for metabolomic analysis, comprising CH (*n* = 6) and TO (*n* = 6). Approximately 30 mg of dorsal muscle tissue was placed into Eppendorf tubes and extracted with a precooled solvent containing an internal standard (methanol: water = 4:1, *v*/*v*). After thorough mixing and low-temperature extraction, the samples were centrifuged at high speed, and the resulting supernatants were collected for subsequent analyses. According to platform requirements, the supernatants were subjected to LC–MS and GC–MS analyses. Quality control (QC) samples were prepared by pooling equal aliquots from all samples to monitor instrument stability and data quality. Liquid chromatography was performed on an ultra-high-performance liquid chromatography (UHPLC, Waters Corporation, Milford, MA, USA) system equipped with a reversed-phase C18 column (2.1 mm × 100 mm, 1.7 μm), with the column temperature maintained at 40 °C. The mobile phases consisted of 0.1% formic acid in water (A) and 0.1% formic acid in acetonitrile (B), with a flow rate of 0.3 mL/min and an injection volume of 2 μL. Metabolites were separated using a gradient elution program. Mass spectrometric detection was conducted on a high-resolution mass spectrometer with an electrospray ionization (ESI) source operated in both positive and negative ion modes, covering a scan range of *m*/*z* 70–1050. Data-dependent acquisition (DDA) was applied to obtain MS/MS spectra for metabolite identification.

For GC–MS analysis, samples were derivatized prior to detection by oximation with 80 μL of methoxyamine hydrochloride in pyridine (15 mg/mL) at 37 °C for 60 min, followed by silylation with 50 μL of BSTFA and 20 μL of hexane at 70 °C for 60 min. GC–MS analysis was performed on a system equipped with an electron ionization (EI) source operating at 70 eV. The ion source temperature was set at 230 °C. Full-scan mode was applied over a mass range of *m*/*z* 50–500. GC separation was carried out on a DB-5MS capillary column (30 m × 0.25 mm × 0.25 μm). High-purity helium (≥99.99%) was used as the carrier gas at a constant flow rate of 1.0 mL/min. A 1 μL aliquot was injected in splitless mode, with an injector temperature of 260 °C. The solvent delay time was set to 5 min. The oven temperature program was as follows: the initial temperature of 60 °C (held for 0.5 min) was ramped to 125 °C at 8 °C/min, to 210 °C at 8 °C/min, to 270 °C at 15 °C/min, and finally to 305 °C at 20 °C/min with a final hold of 5 min. LC–MS analysis was performed using a Waters ACQUITY UPLC I-Class Plus system (Waters Corporation, Milford, MA, USA) coupled to a Thermo Q Exactive mass spectrometer (Thermo Fisher Scientific, Waltham, MA, USA) equipped with a heated electrospray ionization (HESI) source. Data were acquired in both positive and negative ion modes over a mass range of *m*/*z* 70–1050. QC samples were injected at regular intervals throughout the analytical sequence to monitor instrument stability and analytical reproducibility. The processed metabolomic data were subjected to multivariate statistical analyses, including principal component analysis (PCA) and orthogonal partial least squares-discriminant analysis (OPLS-DA), to evaluate metabolic differences between groups. Differentially accumulated metabolites (DEMs) were identified based on VIP > 1, FDR < 0.05 (q-value), and fold change (FC), with the Benjamini–Hochberg method applied to control the false discovery rate in multiple comparisons. FC was defined as the ratio of metabolite abundance in CH to TO. KEGG pathway enrichment analysis was subsequently performed to explore the potential biological functions and regulatory pathways associated with the differential metabolites.

### 2.5. Integrated Transcriptome and Metabolome Analysis

To comprehensively explore the regulatory relationships between transcriptome and metabolome, an integrated multi-omics analysis was conducted. Key module genes obtained from WGCNA and differential metabolites (VIP > 1, (*p* < 0.05), with significant fold changes) were retained for subsequent analysis. All differentially expressed genes (DEGs) and differential metabolites (DEMs) were annotated against the KEGG database, and co-enriched pathways shared by the two datasets were screened to explore the functional association between transcriptional variation and metabolic changes.

For Pearson correlation analysis, only the three fish per group with both transcriptomic and metabolomic data (the overlapping subset of the six metabolomic samples per group) were used to calculate the correlation coefficients between key module genes (DEGs) and differential metabolites (DEMs) within these co-enriched KEGG pathways. Gene–metabolite pairs with |r| > 0.5, *p* < 0.05, and FDR < 0.05 after Benjamini–Hochberg correction were defined as statistically significant. Receiver operating characteristic (ROC) curve analysis was performed using the pROC package in R (v1.19.0.1) to evaluate the diagnostic performance of candidate key genes. The area under the curve (AUC) was calculated, and the optimal cutoff values were determined based on the maximum Youden index. By combining KEGG pathway enrichment, correlation analysis and ROC results, the coordinated variation in genes and metabolites in critical pathways was systematically analyzed to reveal the potential molecular mechanisms underlying transcriptomic and metabolic regulation. Combined with functional annotation, key rate-limiting enzymes or pivotal signaling regulators with significant differential expression, strong correlations with DEMs, reliable diagnostic capacity, and essential biological functions were finally screened and identified as core genes.

### 2.6. RT-qPCR Validation of Key Genes

To validate the transcriptomic data, quantitative real-time PCR (RT-qPCR) was performed to assess the expression levels of six selected key differentially expressed genes. Total RNA extracted from dorsal muscle tissues was reverse-transcribed into first-strand cDNA using a reverse transcription kit (Sangon Biotech, Shanghai, China). Gene-specific primers were synthesized by Sangon Biotech (Shanghai, China), and the sequences are listed in Table 2. RT-qPCR was conducted on a QuantStudio™ 5 Plus Real-Time PCR System (Applied Biosystems, Waltham, MA, USA), with *β-actin* serving as the internal reference gene for normalization. Relative gene expression levels were calculated using the 2^−ΔΔCt^ method. All experiments were performed with three independent biological replicates. Data are presented as mean ± SD, and statistical significance was evaluated using Student’s *t*-test (** *p* < 0.01, *** *p* < 0.001).

## 3. Results

### 3.1. Transcriptome Data Analysis

#### 3.1.1. Transcriptome Assembly and Quality Assessment

**Figure 1:**
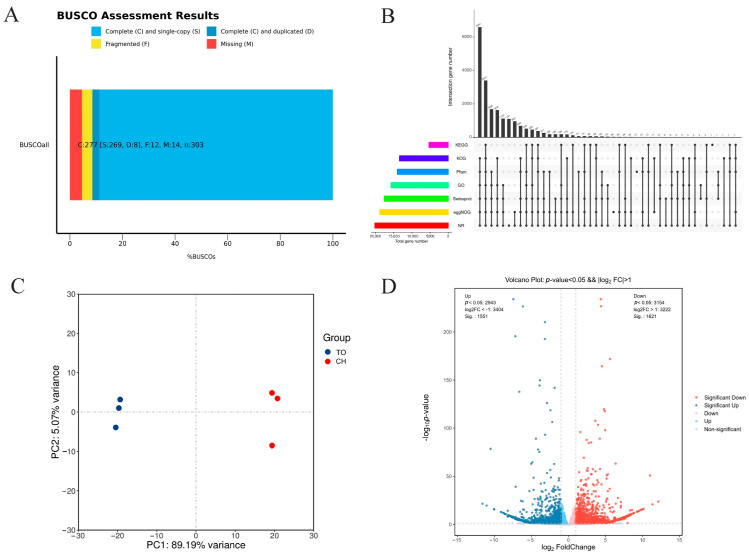
Transcriptome analysis of CH and TO. (**A**). BUSCO assessment of the de novo assembled transcriptome. (**B**). Transcriptome database annotation results. (**C**). Principal component analysis: (**D**). Volcano plot of differentially expressed genes (DEGs).

Transcriptome sequencing was conducted on six samples, generating a total of 40.5 Gb of high-quality clean data, with individual samples yielding between 6.29 and 7.04 Gb. De novo assembly produced 30,351 unigenes, with a total length of 42,212,745 bp and an average length of 1390.82 bp. The length distribution of unigenes indicated good completeness and continuity of the assembly. The proportion of bases with a quality score ≥ Q30 ranged from 95.27% to 95.39%, and the average GC content was 51.82%, demonstrating high sequencing quality suitable for downstream analyses (Table 3). The clean reads were mapped to the assembled unigenes, with mapping rates ranging from 79.41% to 84.47% and minimal variation among samples, indicating that the data met the quality requirements for de novo transcriptome sequencing. The completeness of the assembled transcriptome was further evaluated using BUSCO. A total of 277 (91.4%) BUSCOs were identified as complete, including 269 (88.8%) single-copy and eight (2.6%) duplicated genes, while 12 (4.0%) were fragmented and only 14 (4.6%) were missing (Figure 1A). These results indicate a high-quality and near-complete transcriptome assembly. Functional annotation revealed that 20,204 unigenes (66.57%) were annotated in the NR database, 17,612 (58.03%) in Swiss–Prot, 5369 (17.69%) in KEGG, 13,463 (44.36%) in KOG, 18,848 (62.10%) in egg NOG, 15,731 (51.83%) in GO, and 14,037 (46.25%) in the Pfam database (Figure 1B).

Principal component analysis (PCA) indicated that the first principal component (PC1) explained 89.2% of the total variance. Samples from TO and CH were clearly separated along PC1, and the three biological replicates within each group clustered tightly (Figure 1C), indicating distinct transcriptomic profiles between groups and high reproducibility. Differential expression analysis, using the criteria of |log2FC| ≥ 1 and FDR < 0.05, identified a total of 3172 differentially expressed genes (DEGs), including 1551 upregulated and 1621 downregulated genes (Figure 1D).

#### 3.1.2. WGCNA Analysis

**Figure 2:**
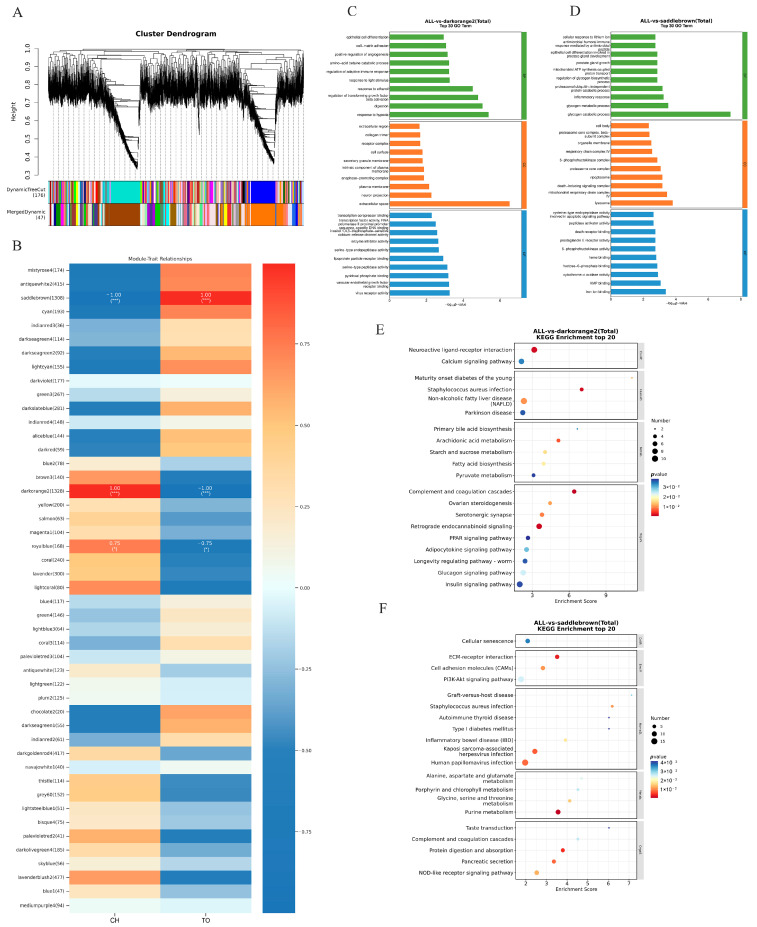
WGCNA and enrichment analysis of CH and TO. (**A**). Gene clustering tree constructed using dissTOM matrix. (**B**) Trait module association heatmap, with vertical axis representing related modules and horizontal axis indicating two varieties; asterisks denote statistical significance (* *p* < 0.05, *** *p* < 0.001). (**C**,**D**). GO enrichment analysis (darkorange2 and saddlebrown modules). (**E**,**F**). KEGG enrichment analysis (darkorange2 and saddlebrown modules).

After preprocessing and filtering the expression data from six samples, a total of 9064 genes were retained for weighted gene co-expression network analysis (WGCNA) and classified into 47 co-expression modules. A topological overlap matrix (TOM) was constructed based on weighted correlation coefficients, and hierarchical clustering was applied to generate the gene dendrogram (Figure 2A). Pearson correlation analysis was used to evaluate the associations between module eigengenes (MEs) and phenotypic traits, with corresponding *p*-values. Modules with an absolute correlation coefficient ≥ 0.3 and *p* < 0.05 were considered significantly associated with the traits. The darkorange2 and saddlebrown modules were identified as key modules showing significant correlations with the studied traits (Figure 2B). Specifically, the darkorange2 module exhibited a strong positive correlation with CH (r = 0.99, *p* < 0.05) and a negative correlation with TO (r = −0.99, *p* < 0.05), whereas the saddlebrown module showed a strong positive correlation with TO (r = 0.99, *p* < 0.05) and a negative correlation with CH (r = −0.99, *p* < 0.05), indicating opposite transcriptional co-expression patterns between the two groups (Figure 2B).

To explore the potential functional roles of these modules, genes within darkorange2 and saddlebrown were subjected to Gene Ontology (GO) and KEGG pathway enrichment analyses. GO enrichment of the darkorange2 module revealed that DEGs were predominantly involved in growth- and development-related processes. At the Biological Process (BP) level, DEGs were mainly associated with glycogen metabolism and degradation, mitochondrial ATP synthesis, and protein degradation. In the Cellular Component (CC) category, genes were enriched in the mitochondrial respiratory chain complex, proteasome core complex, and lysosome. At the Molecular Function (MF) level, DEGs were primarily linked to glycolysis-related enzymatic activities and oxidative phosphorylation (Figure 2C), indicating that genes in CH are closely associated with energy metabolism and likely play key roles in growth and development.

KEGG pathway enrichment analysis further revealed that DEGs in CH were enriched in signaling and energy metabolism pathways, including fatty acid biosynthesis, pyruvate metabolism, PPAR signaling, insulin signaling, and neuroactive ligand–receptor interactions (Figure 2E), suggesting that CH growth is closely regulated by multiple metabolic and signal transduction pathways.

In contrast, GO enrichment of the saddlebrown module showed that DEGs were significantly enriched in biological processes related to hypoxia response, angiogenesis, digestion, and immune regulation (Figure 2D). At the CC level, genes were mainly associated with the extracellular space and plasma membrane. MF analysis indicated enrichment in receptor activity, serine-type protease activity, and transcriptional regulation, implying roles in environmental stress adaptation and signal transduction. KEGG pathway enrichment revealed that genes in the saddlebrown module were enriched in immune-related and cell structure-associated pathways, including ECM–receptor interaction, cell adhesion molecules (CAMs), NOD-like receptor signaling, and cellular senescence (Figure 2F), suggesting that this module contributes to immune response regulation and cellular homeostasis.

### 3.2. Metabolomics Analysis

#### 3.2.1. Metabolite Identification and Statistical Analysis

**Figure 3:**
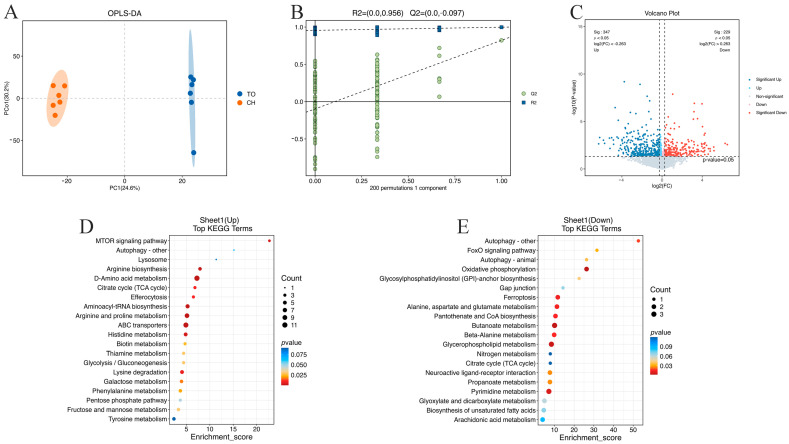
Metabolome analysis of CH and TO. (**A**). Orthogonal Partial Least Squares-Discriminant Analysis (OPLS-DA). (**B**). Permutation Test Plot of OPLS-DA. (**C**). Metabolome Volcano Plot. (**D**,**E**). Enrichment analysis of up- and downregulated metabolites in the metabolome.

Non-targeted metabolomic analysis using LC–MS/MS and GC–MS detected a total of 2931 metabolite features. OPLS-DA revealed a clear separation between TO and CH along the predictive component, indicating significant metabolic differences between the two groups and demonstrating strong discriminative performance of the model (Figure 3A). Permutation tests confirmed that the Q^2^ intercepts of the OPLS-DA model were all below zero, suggesting that the model was not overfitted and possessed robust predictive ability and reliability (Figure 3B). Differentially accumulated metabolite analysis identified 576 significantly altered DEMs, of which 347 were upregulated and 229 downregulated in CH compared to TO (Figure 3C).

#### 3.2.2. KEGG Enrichment Analysis of Differential Metabolites

KEGG enrichment analysis showed that the upregulated differential metabolites were primarily enriched in pathways related to energy metabolism, amino acid metabolism, and autophagy-associated transport processes. Key enriched pathways included the mTOR signaling pathway, Autophagy—other, Lysosome, Citrate cycle (TCA cycle), and Glycolysis/Gluconeogenesis. Additionally, multiple amino acid metabolism pathways were significantly enriched, such as arginine biosynthesis, arginine and proline metabolism, histidine metabolism, phenylalanine metabolism, and tyrosine metabolism (Figure 3D). These results suggest that the upregulated metabolites predominantly contribute to energy production, amino acid metabolic regulation, and autophagy-related processes, potentially supporting the enhanced metabolic activity underlying the rapid growth of CH.

In contrast, downregulated metabolites were mainly enriched in pathways associated with autophagy regulation, oxidative phosphorylation, lipid metabolism, and signal transduction, including Autophagy—other, Oxidative phosphorylation, FoxO signaling pathway, Ferroptosis, and several lipid-related pathways (Figure 3E). These findings indicate that specific metabolic processes may be suppressed or remodeled in CH, reflecting distinct metabolic adaptations between the two groups.

### 3.3. Integrated Analysis of Transcriptome and Metabolome

**Figure 4:**
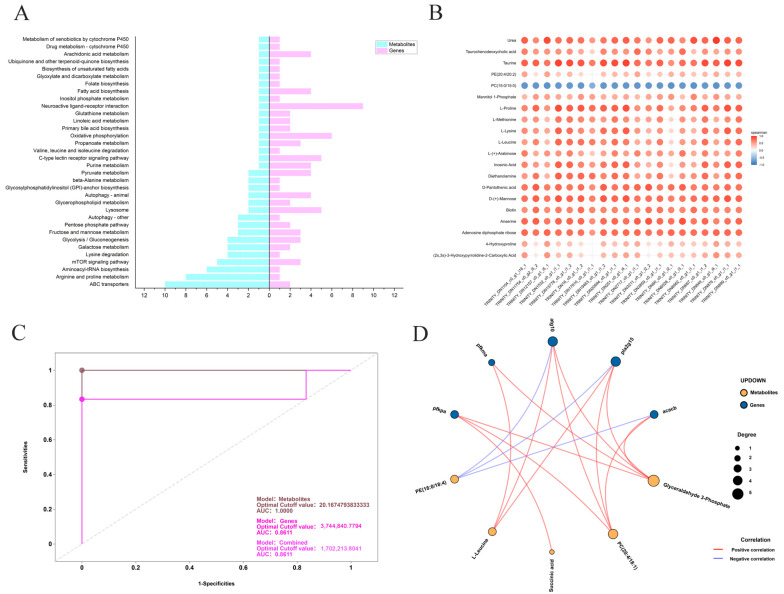
Transcriptome–metabolome integrated analysis of CH. (**A**). Co-expression analysis results. Vertical axis: enriched metabolites; horizontal axis: key module genes. (**B**). Genes and metabolites sharing common pathways. (**C**). ROC curve analysis of key genes and metabolites. (**D**). Co-expression analysis of key genes and metabolites.

To investigate the relationship between transcriptomic and metabolomic alterations, KEGG pathways enriched in key WGCNA modules were integrated with pathways identified through metabolomic analysis. Integrated pathway analysis revealed that the commonly enriched pathways in the fast-growing CH group were mainly associated with growth and metabolic regulation. These pathways included glycolysis, the MAPK signaling pathway, the mTOR signaling pathway, aminoacyl-tRNA biosynthesis, and multiple lipid metabolism pathways. A total of 34 commonly enriched pathways was identified across the two datasets, involving 82 genes and 80 metabolites (Figure 4A).

Based on the genes and metabolites involved in these shared pathways, a co-expression network was constructed to evaluate coordinated changes between genes and metabolites (Figure 4B). After filtering using the criteria of |r| > 0.5, *p* < 0.05, and FDR < 0.05, 26 genes and 31 metabolites met the screening thresholds and were identified as candidate key genes and metabolites. To assess the discriminative power of these candidate genes and metabolites between the two experimental groups, receiver operating characteristic (ROC) curve analysis was performed (Figure 4C). The metabolite-based model exhibited excellent performance, with an AUC value of 1.000, indicating perfect separation between the two groups. The gene-based model achieved an AUC of 0.8611, demonstrating good diagnostic accuracy. These findings suggest that the identified candidate genes and metabolites possess strong potential as biomarkers for distinguishing the experimental groups.

Further functional annotation of the candidate genes, together with evaluation of their potential roles as rate-limiting enzymes or key signaling regulators, identified the following key genes: *pfkpa*, *pfkma*, *sesn2*, *atg10*, *pla2g15*, and *acacb*. Representative metabolites included glyceraldehyde-3-phosphate (G3P), L-leucine, phosphatidylcholine (PC), phosphatidylethanolamine (PE), and succinate. Correlation network analysis of these key genes and metabolites is shown in Figure 4D. Within the glycolytic pathway, G3P showed significant positive correlations with *pfkpa* (r = 0.89, *p* < 0.001) and *pfkma* (r = 0.92, *p* < 0.001). In addition, succinate, a downstream metabolite in the tricarboxylic acid (TCA) cycle, was positively correlated with *pfkpa* (r = 0.61, *p* < 0.05). In lipid metabolism, *acacb* was positively correlated with PC (20:4/18:1) (r = 0.70, *p* < 0.05) and negatively correlated with PE (18:0/18:4) (r = −0.63, *p* < 0.05), highlighting the coordinated regulatory effects of key metabolic genes on lipid metabolites.

To further characterize the expression patterns of these key genes, FPKM values from all samples in the CH and TO groups were compared (Table 4). The results showed that several key genes, including *pfkpa*, *pfkma*, *pla2g15*, and *acacb*, exhibited higher expression levels in the CH group than in the TO group. Notably, *acacb* expression was extremely low or undetectable in the TO group, whereas measurable expression levels were maintained in the CH group. In contrast, genes such as *sesn2* and *atg10* displayed relatively small or non-significant differences between the two groups. Overall, these expression patterns support the differential transcriptional regulation of metabolism-related pathways between the experimental groups.

### 3.4. RT-qPCR Validation

**Figure 5:**
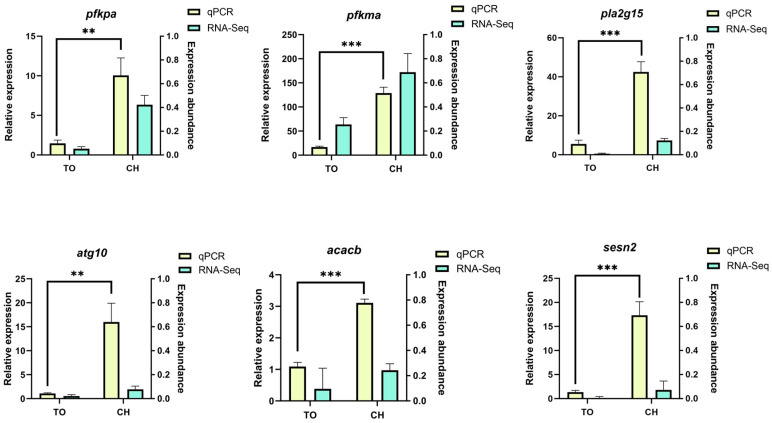
Validation of RNA-seq data by RT-qPCR. Relative mRNA expression levels of key growth-related genes. Data are presented as mean ± SD (*n* = 3 biological replicates). Statistical significance was determined using Student’s *t*-test (** *p* < 0.01, *** *p* < 0.001). qPCR data were normalized using the 2^−ΔΔCt^ method with *β-actin* as the reference gene.

To validate the reliability of the transcriptomic data, six key differentially expressed genes (*pfkpa*, *pfkma*, *pla2g15*, *acacb*, *atg10*, and *sesn2*) were selected for RT-qPCR analysis. The RT-qPCR results showed expression trends consistent with those obtained from RNA-seq analysis, with all genes exhibiting upregulation in the CH group compared to the TO group (Figure 5). However, differences in the magnitude of fold change were observed between the two methods. Overall, the consistency in expression trends supports the reliability of the transcriptomic data.

## 4. Discussion

### 4.1. Energy Metabolism Remodeling, Growth Signal Activation, and Enhanced Tissue Development Regulation at the Transcriptional Level of CH

Transcriptional regulation is a fundamental driver of metabolic pathway activity, as it modulates the expression of key metabolic enzymes and signaling molecules, thereby influencing the generation, allocation, and efficiency of energy utilization at the source [25]. In the present study, transcriptomic analysis revealed that differentially expressed genes were significantly enriched in energy metabolism-related pathways, including the insulin signaling pathway, PPAR signaling pathway, and pyruvate metabolism, as well as glucose- and lipid-associated pathways such as starch and sucrose metabolism and fatty acid biosynthesis (Figure 2C,E).

These pathways have been widely recognized as central regulatory networks for energy supply and utilization. The insulin signaling pathway, for example, plays a critical role in maintaining cellular energy balance and regulating growth rate [26]. The PPAR signaling pathway promotes growth and development by modulating the expression of genes involved in cell proliferation, differentiation, and cell cycle progression [27]. Glycogen metabolism, as a fundamental process for energy storage and supply, not only provides a readily accessible energy source but also influences cell differentiation and the activity of multiple signaling pathways [28]. Pyruvate, the end product of glycolysis, enters the tricarboxylic acid (TCA) cycle within mitochondria, serving as a precursor for oxidative phosphorylation and efficient ATP production, while also supplying carbon skeletons for various biosynthetic pathways [29]. Additionally, fatty acid biosynthesis pathways are intimately linked with cell proliferation, growth, and development, particularly under conditions of rapid cell division such as in stem cells, developing tissues, or tumor cells [30].

Interestingly, KEGG enrichment analysis of the CH transcriptome revealed significant enrichment of pathways that are fundamental to growth and development. Further examination of the genes within these pathways identified *mapk8a*, *acacb*, and *pkmb* as closely associated with growth and developmental processes, suggesting their potential roles in regulating rapid growth.

The JNK-family kinase *mapk8a*, acetyl–CoA carboxylase beta (*acacb*), and pyruvate kinase M isoform B (*pkmb*) were significantly upregulated in CH, forming a putative mechanism that may underpin its rapid growth phenotype. *Mapk8a* integrates extracellular signals through the MAPK cascade to regulate cell proliferation and differentiation, while interfacing with mTOR and AMPK signaling to coordinate energy sensing with anabolic growth [31,32]. *Acacb* catalyzes the conversion of acetyl–CoA to malonyl–CoA, a central metabolic node that regulates fatty acid oxidation and feeds back to AMPK/mTOR signaling, linking cellular energy status to growth demands [33,34]. *Pkmb*, a rate-limiting glycolytic enzyme, also functions as a nuclear coactivator, interacting with transcription factors such as HIF-1α and β-catenin to synchronize energy production with cell cycle progression and the expression of proliferation- and metabolism-related genes [35]. The coordinated upregulation of these genes in CH suggests a transcriptionally driven mechanism that integrates energy metabolism, signaling, and developmental programs, contributing to the hybrid’s exceptional growth.

### 4.2. Enhanced Energy Metabolism, Amino Acid Metabolism, and Energy Sensing Capabilities in CH

In the present metabolomic study, differential metabolites in the CH group were significantly enriched in the mTOR signaling pathway, glycolysis/gluconeogenesis, the tricarboxylic acid (TCA) cycle, and amino acid metabolism. Although some mechanistic evidence is derived from mammalian studies, these signaling pathways are highly conserved across vertebrates. Therefore, mammalian findings were used as references to support functional interpretation in fish in the absence of sufficient species-specific evidence. These pathways are closely associated with energy supply and nutrient sensing and have been shown to play pivotal roles in regulating growth and development across diverse animal species.

The mTOR signaling pathway is an evolutionarily conserved nutrient-sensing hub present in nearly all eukaryotes, integrating extracellular signals to regulate cell growth, metabolism, and tissue development [36]. In the liver of *Micropterus salmoides*, mTOR not only regulates metabolism but is also closely linked to growth performance, feed efficiency, and energy homeostasis. The activation of mTOR enhances specific growth rate (SGR) and weight gain by upregulating glycolysis, the TCA cycle, and insulin secretion, while reducing hepatic lipid accumulation, thereby promoting overall growth [37]. In zebrafish (*Danio rerio*), leucine has been reported to regulate the mTOR signaling pathway by increasing the phosphorylation of downstream effectors such as S6K1 and S6, enhancing protein synthesis and growth-related metabolic processes [38]. The elevated leucine levels observed in CH, together with its known role as an upstream regulator of mTORC1 in vertebrates, support that amino acid-dependent signaling contributes to the modulation of growth-related pathways. This inference is consistent with the observed growth advantage of CH.

Glycolysis and the TCA cycle are central to cellular energy metabolism, directly influencing growth and development. Glycolysis rapidly generates ATP and produces pyruvate as a substrate for the TCA cycle [39], which, through oxidative metabolism, generates NADH and FADH_2_ to supply reducing equivalents for oxidative phosphorylation, thereby maximizing ATP production [40]. In fish, increased activity of these pathways improves feed efficiency and growth performance. For example, in largemouth bass, an appropriate carbohydrate source activated glycolytic and TCA cycle enzymes and associated gene expression, enhancing glucose metabolism and significantly increasing weight gain, demonstrating that the upregulation of energy metabolism pathways supports growth advantages [41]. In CH, differential metabolites were significantly enriched in glycolysis and the TCA cycle, indicating the activation of these energy pathways, which likely provides sufficient energy to enhance growth performance and feed utilization efficiency.

Amino acid metabolism not only supplies precursors for protein synthesis but also plays critical roles in energy metabolism, hormone production, cell signaling, and antioxidant defense [42]. Arginine metabolism, for example, has been shown to support protein synthesis and growth by modulating energy metabolism and TOR signaling, thereby affecting growth, nutrient utilization, endocrine regulation, and immune function in fish. Other studies have similarly highlighted the close link between amino acid metabolism and overall energy homeostasis, supporting the coordination between metabolic state and growth [43]. In the present study, CH exhibited significant enrichment in multiple amino acid metabolism pathways, including arginine, leucine, and ornithine, with higher metabolite concentrations than TO. Elevated amino acid levels likely provide abundant protein precursors for growth and simultaneously regulate energy metabolism and cell signaling, thereby contributing to the enhanced growth performance of CH.

### 4.3. Synergistic Regulatory Network of Growth and Development in the Growth Advantage of CH

In this study, we integrated transcriptomic and metabolomic analyses to elucidate the potential molecular regulatory mechanisms underlying the rapid growth of CH (Figure 5). The integrative analysis identified the mTOR signaling pathway as a central hub, linking multiple key pathways and playing a pivotal regulatory role in growth and metabolism.

First, several key energy metabolism pathways, including glycolysis, the pentose phosphate pathway, pyruvate metabolism, and oxidative phosphorylation, were significantly enriched in CH, indicating an overall enhancement of cellular energy metabolism. Within glycolysis, the rate-limiting enzyme genes *pfkpa* and *pfkma* were significantly upregulated and showed strong positive correlations with the key intermediate metabolite glyceraldehyde-3-phosphate (G3P) (Figure 4D), suggesting increased glycolytic flux to provide sufficient substrates for energy production and biosynthetic processes.

*Pfk-1*, as a rate-limiting enzyme in glycolysis, catalyzes the conversion of fructose-6-phosphate to fructose-1,6-bisphosphate, serving as a central regulatory node in cellular energy supply and metabolic control [44]. Its activity is tightly regulated by cellular energy status (ATP/AMP ratio), hormonal signals, and multiple signaling pathways, and is closely associated with cell proliferation and adaptation to energy demands [45]. G3P, generated downstream of *pfk-1* during the cleavage phase of glycolysis, serves as a substrate for GAPDH to support ATP production and biosynthetic processes [29,46]. The positive correlation observed between *pfk-1* expression and G3P abundance indicates the coordinated enhancement of glycolysis at both transcriptional and metabolic levels, supporting an overall increase in glycolytic flux.

Succinate also showed a positive correlation with *pfkpa*. Although a direct regulatory relationship between succinate and *pfkpa* has not been reported, previous studies indicate that succinate can activate the MAPK signaling pathway [47]. In fish, the MAPK/Erk axis has been shown to promote glycolysis [48], and the 6-phosphofructo-2-kinase/fructose-2,6-bisphosphatase (*pfkfb*) system that generates the potent allosteric activator Fru-2,6-P_2_ for *pfk-1* is conserved in teleosts [29]. Given that MAPK signaling has been reported to regulate *pfk-1* activity indirectly via the PFKFB3-mediated control of Fru-2,6-P_2_ levels in other vertebrate systems, a similar mechanism may function in fish, suggesting that MAPK activation could potentially enhance *pfkpa* activity [49]. Coordinated enhancement of glycolysis and mitochondrial oxidative metabolism increases cellular ATP, NADH, and NADPH supply [50]. Improved energy status decreases the AMP/ATP ratio, suppressing AMPK-mediated energy stress signaling, and relieving its inhibitory effect on mTORC1 [51]. This correlation likely reflects functional coordination between glycolytic activation and mitochondrial metabolism rather than a direct regulatory effect. These metabolic conditions may create a favorable intracellular environment for the potential activation of mTORC1 signaling.

In addition to energy metabolism, the integrated analysis highlighted the role of leucine in mTORC1 activation. Although transcriptional correlation between leucine and the key mTOR regulator *sesn2* was weak, previous studies have shown that *sesn2* binds leucine directly, undergoing conformational changes that relieve the inhibition of mTORC1 via the GATOR complex [52]. Thus, leucine primarily regulates mTORC1 at the protein level.

The fatty acid biosynthesis pathway was also significantly enriched, and mTORC1 activation may promote fatty acid and membrane lipid synthesis via the SREBP pathway [53]. Within this pathway, *acacb* was significantly correlated with several phospholipid metabolites. Acetyl–CoA carboxylase (*acc*, including *acacb*) catalyzes the conversion of acetyl–CoA to malonyl–CoA, which is the rate-limiting step of de novo fatty acid synthesis, determining the size of the intracellular acyl-CoA pool [54]. Malonyl–CoA subsequently enters fatty acid synthase reactions to generate long-chain fatty acids, which serve as carbon donors for complex lipids, including the side chains of phosphatidylcholine (PC) and phosphatidylethanolamine (PE) [55]. Previous studies have shown that the inhibition of *acc* activity alters phospholipid composition, indicating that *acc* controls complex lipid synthesis via the regulation of the fatty acid pool [56].

In CH, the relative increase in PC and decrease in PE suggest a metabolic shift toward structural membrane lipid synthesis, likely reflecting enhanced membrane biogenesis. Correlation analysis revealed that PE was negatively associated with the autophagy-related factor *atg10* and the lysosomal phospholipid hydrolase *pla2g15*, whereas PC was positively correlated with *atg10*. PE is a key component of autophagosomal membranes, and its abundance reflects autophagy-associated membrane dynamics [57]. Under mTORC1 activation, reduced autophagic flux coupled with enhanced membrane lipid synthesis indicates a metabolic transition from a “resource recycling” state to a “growth expansion” state, facilitating membrane system expansion during rapid growth and development [58,59].

**Figure 6:**
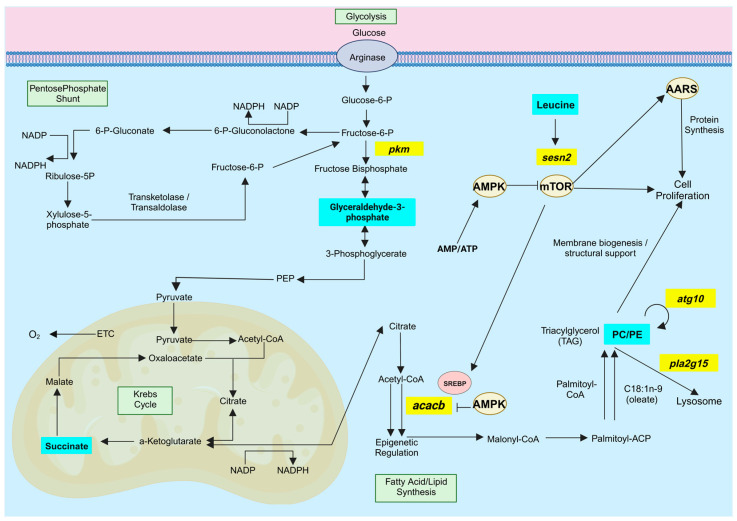
Proposed transcriptional–metabolic coupling mechanism in this study. Enhanced energy metabolism inhibits AMPK signaling, while leucine activates mTORC1 via the *sesn2*–gator pathway, synergistically promoting lipid synthesis and protein synthesis to accelerate muscle growth. Yellow rectangles indicate key genes; blue rectangles indicate key metabolites; light brown ellipses represent core metabolic pathways.

Based on these integrated results, a hypothetical transcription–metabolic regulatory network was constructed (Figure 6). It should be noted that this regulatory model is primarily based on muscle transcriptomic data combined with metabolomic profiling, and therefore reflects tissue-level regulatory responses rather than systemic metabolic regulation across all organs. Enhanced glycolysis, the pentose phosphate pathway, pyruvate metabolism, and oxidative phosphorylation provide sufficient energy, reducing the intracellular AMP/ATP ratio. This suppresses AMPK-mediated energy stress signaling, potentially contributing to sustained mTORC1 activation. Concurrently, metabolomic analysis showed significant upregulation of key amino acids, especially leucine, which directly activates mTORC1 via the *sesn2*-GATOR complex, amplifying mTORC1 signaling. Activated mTORC1 integrates energy status with amino acid signals to promote fatty acid synthesis and membrane lipid biogenesis, providing the structural basis for cell proliferation and protein synthesis. These coordinated transcriptional and metabolic changes provide a mechanistic explanation for the observed heterosis in CH. Unlike single-gene effects, hybrid vigor in this hybrid appears to arise from coordinated molecular reprogramming, characterized by enhanced energy metabolism, improved nutrient sensing, and coordinated anabolic activity. The integration of transcriptional regulation and metabolic flux may therefore contribute to improved energy efficiency and biosynthetic capacity, ultimately driving the superior growth performance of the hybrid.

Collectively, these results support the existence of a synergistic regulatory network, characterized by enhanced energy metabolism, active amino acid sensing, and lipid metabolic remodeling. Given that liver-specific data were not included in the present study, the proposed mechanism should be interpreted as a muscle-centered metabolic and transcriptional regulatory framework rather than a whole-organism metabolic model. The coordinated transcriptional and metabolic alterations observed are highly consistent with the growth advantage of CH, although further functional validation is required to fully elucidate the underlying molecular mechanisms.

## 5. Conclusions

This study reveals that the rapid growth performance of CH is driven by a coordinated transcriptional and metabolic regulatory network centered on energy metabolism and nutrient sensing. Multi-omics analysis showed that key pathways related to glycolysis, the TCA cycle, amino acid metabolism, and lipid biosynthesis were significantly enhanced in CH. At the transcriptional level, key genes including *mapk8a*, *acacb*, and *pkmb* were upregulated, suggesting strengthened energy utilization and growth-related signaling. Metabolomic data further indicated elevated levels of amino acids, particularly leucine, and activation of the mTOR signaling pathway.

Integrated analysis suggests that mTORC1 acts as a central hub linking energy status, amino acid availability, and lipid metabolic remodeling based on integrative omics evidence. Enhanced energy production reduces cellular energy stress and promotes sustained mTORC1 activation, thereby stimulating protein synthesis, membrane biogenesis, and cell proliferation. Collectively, these findings suggest that the synergistic regulation of energy metabolism, amino acid sensing, and lipid metabolism underlies the superior growth phenotype of CH, providing new insights into the molecular mechanisms of growth regulation in fish. However, further functional studies are required to validate the causal roles of these pathways in mediating hybrid growth advantage.

## Figures and Tables

**Table 1:** Monthly body weight (g, mean ± SD) of Chenhai No.1 and *T. ovatus* [9].

Fish Type	May	June	July	August
*T. ovatus*	26.55 ± 1.69	110.14 ± 6.14	234.97 ± 15.36	427.88 ± 23.60
Chenhai No.1	33.66 ± 1.83	138.94 ± 11.48 *	329.00 ± 33.77 *	579.87 ± 36.98 *

The * showed significant associations with growth traits (*p* < 0.05).

**Table 2:** Primers used for RT-qPCR validation.

Gene	Forward Primer (5′ → 3′)	Reverse Primer (5′ → 3′)
*pfkpa*	CTGAGCAGATGCGAATGCAC	TTCCTGCTGTCGAACACTCC
*pfkma*	GAGCTGCCACACTCTGTTCT	CTGCCGATAACGGTACCTCC
*pla2g15*	GATTGGAGGAAAGCCCCGAA	CCGAGTAGAGCCGCTTGTAG
*acacb*	GCTCCCCTTTGCAGTTTTGG	TTCACGTGCATGGCTACTGT
*atg10*	CAGTCAGTCAGCAGGAGCAT	AGTTCACTGGTCTGTGCTGG
*sesn2*	GCAAGAGATGGCCGGTAACT	GCCTCGCCTAAGCTTTTCACG

**Table 3:** RNA-seq results of muscle tissues from CH and TO.

Sample	Raw Reads	Raw Bases	Clean Reads	Clean Bases	Valid Bases (%)	Q30 (%)	GC (%)
CH_1	48,940,000	7,240,000,000	47,560,000	7,040,000,000	97.16	95.35	51.84
CH_2	48,040,000	7,110,000,000	46,650,000	6,900,000,000	97.10	95.37	52.02
CH_3	48,210,000	7,130,000,000	46,780,000	6,920,000,000	97.05	95.39	51.78
TO_1	44,000,000	6,490,000,000	42,620,000	6,290,000,000	96.86	95.27	51.75
TO_2	44,800,000	6,610,000,000	43,360,000	6,400,000,000	96.79	95.30	51.74
TO_3	48,460,000	7,160,000,000	47,000,000	6,950,000,000	96.98	95.33	51.82

**Table 4:** FPKM values of core genes in different groups.

Gene Name	CH1	CH2	CH3	TO1	TO2	TO3
*pfkpa*	7.200458	4.996937	6.819695	1.050288	0.790558	0.535896
*pfkma*	140.5003	159.9265	215.3718	78.98687	60.50867	51.13410
*pla2g15*	7.265181	6.258794	8.378650	0.207358	0.792720	0.538681
*acacb*	2.458238	2.135175	2.734112	0.000000	0.796792	0.000000
*atg10*	1.320900	2.641712	1.870870	0.579426	0.288811	0.817520
*sesn2*	3.682760	1.743272	0.000000	0.484291	0.001953	0.000000

## Data Availability

The complete clean reads for the libraries used in this study have been uploaded to the NCBI Sequence Read Archive (SRA) site (http://www.ncbi.nlm.nih.gov/sra/; BioProject ID: PRJNA1445519, accessed on 12 June 2026).

## Abbreviations

The following abbreviations are used in this manuscript:

CH	Hybrid golden pompano “Chenhai No. 1”
TO	*Trachinotus ovatus*
DEGs	Differentially Expressed Genes
*pfkpa*	Phosphofructokinase, Platelet type
*pfkma*	Phosphofructokinase, Muscle type
*acacb*	Acetyl–CoA Carboxylase Beta
*pkmb*	Pyruvate Kinase M Isoform B
*sesn2*	Sestrin 2
*atg10*	Autophagy-Related 10
*pla2g15*	Phospholipase A2 Group XV
PC	Phosphatidylcholine
PE	Phosphatidylethanolamine
G3P	Glyceraldehyde-3-phosphate
